# Thoracic myelopathy caused by an extremely rare aberrant epidural ligament: A case report

**DOI:** 10.1097/MD.0000000000017344

**Published:** 2019-10-11

**Authors:** Takashi Hirai, Toshitaka Yoshii, Takahiro Tanimoto, Shuta Ushio, Shinichi Sasaki, Hiroyuki Inose, Masato Yuasa, Atsushi Okawa

**Affiliations:** a Department of Orthopedic Surgery, Tokyo Medical and Dental University; b Department of Orthopedic Surgery, Nerima General Hospital, Tokyo, Japan.

**Keywords:** anatomical normality, epidural fibrous band, heterotopic ligament, lipomatosis, thoracic myelopathy

## Abstract

**Rationale:**

The meningovertebral ligaments are a group of tissues that connect the dura and the vertebral bone. Abnormal fibrous ligaments in the canal space, which are essentially different from these ligaments, have been identified and their presence very rarely results in spinal disorder.

**Patient concerns:**

A 20-year-old Mongolian woman had developed persistent headache at 15 years of age. She then became unable to run fast when she was 19 years old and had progressively declining ability to move. She complained of back pain and unstable gait 6 months prior to presentation. Physical examination revealed exaggerated deep tendon reflexes in the lower extremities and decreased proximal leg muscle strength bilaterally.

**Diagnoses:**

Magnetic resonance imaging (MRI) revealed abnormal bands compressing the spinal cord at the T10/11 level, with large epidural lipomatosis dorsal to the dural tube.

**Intervention:**

To decompress the cord, posterior laminectomy for T3-L3 and removal of the heterotopic ligaments were performed with T8-L1 posterior fusion.

**Outcomes:**

Sufficient decompression of the cord was noted on postoperative MRI at the affected segments. The patient could subsequently walk without a cane and headache resolved immediately after the operation.

**Lessons:**

The presence of an aberrant epidural band is a rare pathologic state that often coexists with a surrounding lipomatosis and can lead to spinal cord compression. Removal of the band is a promising treatment for myelopathy caused by the compressive lesion.

## 1 Introduction

Thoracic myelopathy caused by abnormal epidural ligaments is an extremely rare condition. The meningovertebral ligaments lie in the spinal canal and connect the dural sac with the vertebral bone or the posterior longitudinal ligament.^[1]^ Although these ligaments generally consist segmentally of ventral and lateral fibrous bands, abnormal epidural ligaments are quite different. The aberrant ligament runs continuously from the cervical spine to the sacrum and causes severe compression of the spinal cord and is accompanied by excessive epidural fat deposition.

Here, we report a case of myelopathy caused by heterotopic epidural ligament and discuss the clinical and pathological characteristics of this unusual condition.

## 2 Case report

This case report was approved by the ethics committee of Tokyo Medical and Dental University Hospital. Informed consent was obtained from the patient for publication of this case report and accompanying images.

A 20-year-old Mongolian woman developed persistent headache when she was 15 years old. By 19 years of age, she was unable to run fast and had progressively declining ability to move. She subsequently complained of back pain and unstable gait 6 months prior to presentation. There was no history of spinal disorder. Physical examination revealed exaggerated deep tendon reflexes in the lower extremities with decreased proximal leg muscle strength bilaterally. There was decreased sensation in the lower extremities bilaterally.

## 3 Results

### 3.1 Radiologic findings

**Figure 1:**
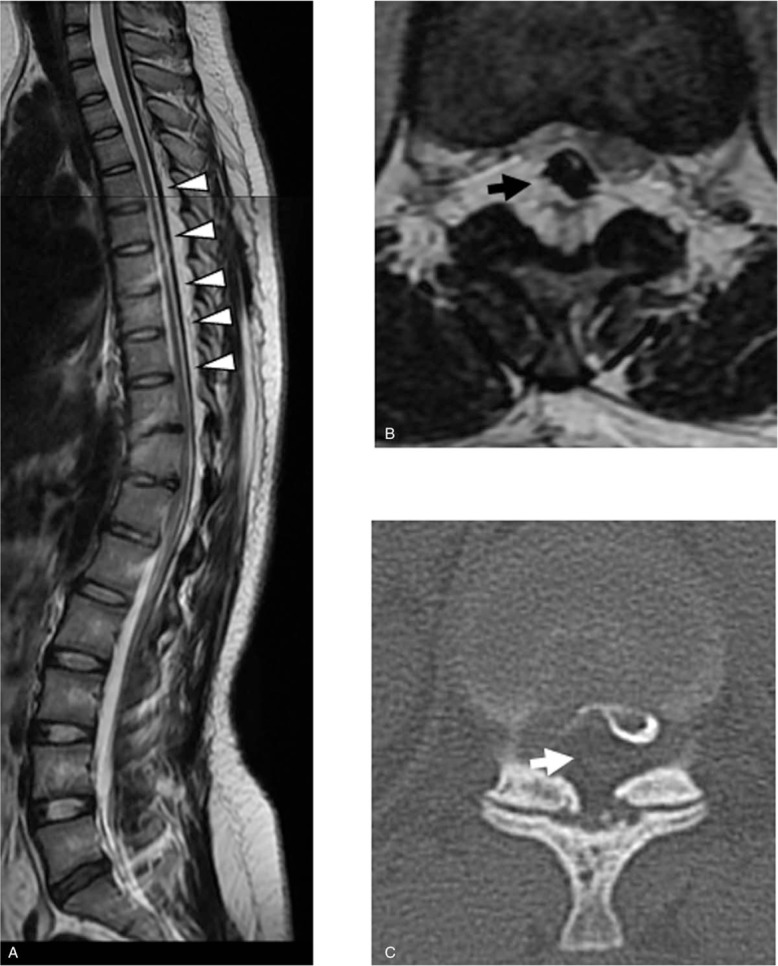
Preoperative magnetic resonance imaging. (A) Epidural ligament running from the cervical to lumbar spine observed on sagittal view (white arrowhead). (B) Fibrous band severely compressing the spinal cord (black arrow). (C) Thick fibrous ligament confirmed in the epidural space (white arrow).

Magnetic resonance imaging (MRI) demonstrated epidural fat deposition and an abnormal fibrous band was seen connecting the dural sac to the thoracic spine (Fig. 1A, B). This abnormal fibrous band severely compressed the spinal cord at the T10-T11 level with resulting hyperkyphosis. The presence of the abnormal fibrous ligament in the epidural space was also confirmed on computed tomography after myelography (Fig. 1C).

### 3.2 Surgical treatment

**Figure 2:**
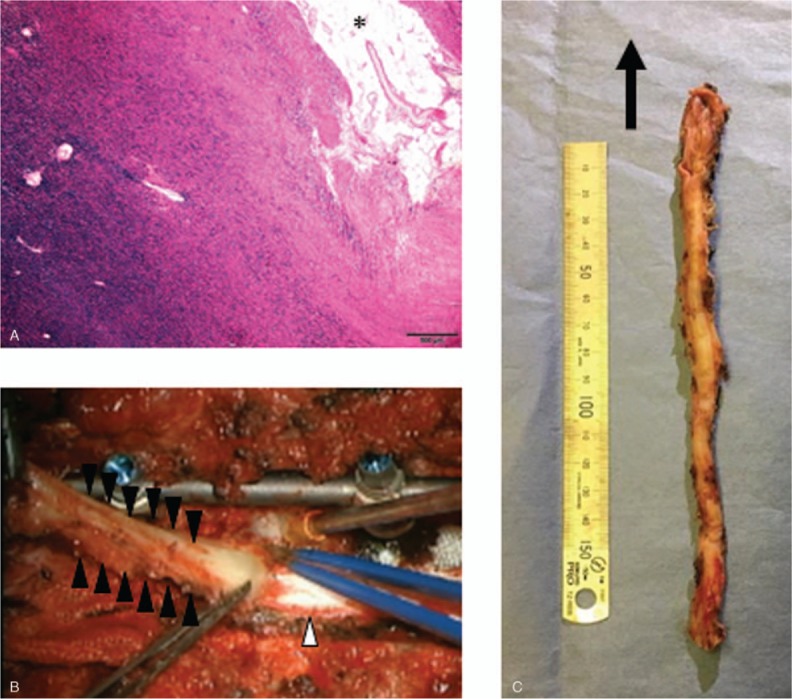
(A) Histology of fibrous epidural ligament (elastic fibers with fat tissue [∗]). (B) Intraoperative photo showing the fibrous epidural ligament (black arrowhead) continuous with the dural sac dorsally (white arrowhead). (C) Whole body of the abnormal ligament (arrow direction is rostral).

Partial laminectomy at L3 and biopsy were performed prior to decompression surgery. Histological examination revealed that the abnormal band differentially consisted of elastic fibers (Fig. 2A). Laminectomy was performed for T3 to L3. Posterior instrumentation at T8 to L1 was also applied to prevent postoperative kyphotic change. A large amount of fat tissue was noted posterior to the fibrous band following laminectomy. The epidural band completely covered the dura and was continuous with the dural sac. This abnormal ligament was tight and adherent to the pedicle of the vertebral bone segmentally. The ligament was peeled off from inferior to superior after cutting the segmental attachment bilaterally (Fig. 2B). Fortunately, the ligament was not adherent to the dura mater. Approximately 23 cm of fibrous structure was resected (Fig. 2C). Spinal cord decompression was confirmed using intraoperative sonography.

### 3.3 Postoperative course

Spasticity in the lower extremities gradually improved by 6 months after surgery. Numbness in the lower extremities remained but had improved. The gait disturbance disappeared. Headache also improved. However, she complained of mild low back pain at 2 years after surgery. Postoperative MRI revealed that the cord compression and epidural band had disappeared.

### 3.4 MRI of the parents

**Figure 3:**
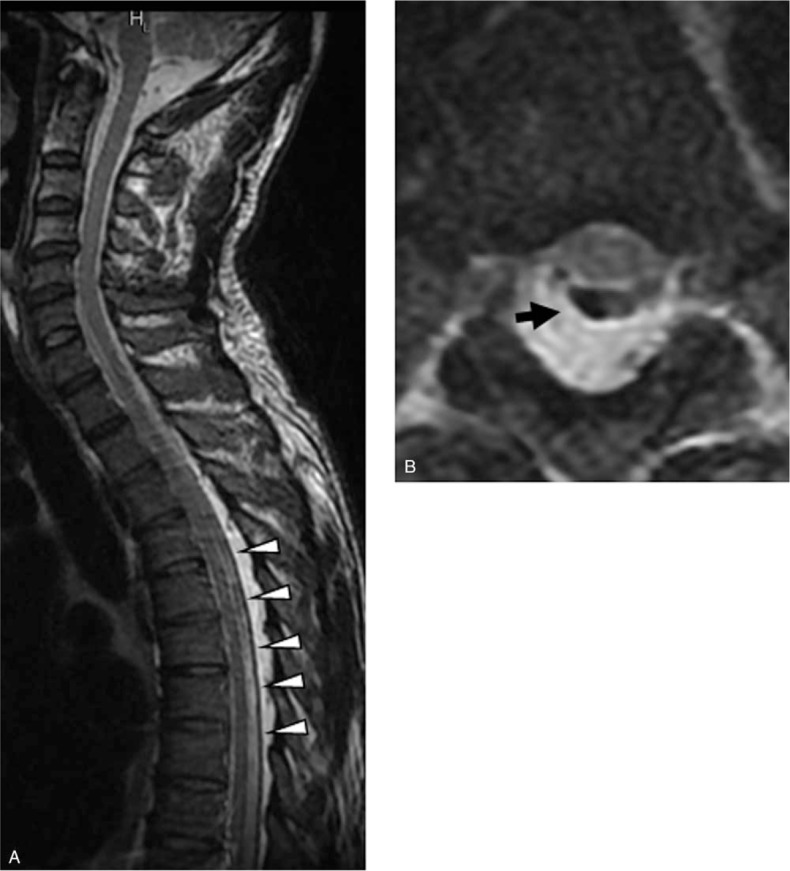
Magnetic resonance imaging of the patient's father. (A) Fibrous epidural ligament (white arrowhead) with dorsal epidural fat tissue deposition. (B) The abnormal ligament observed on axial image (black arrow).

Her parents voluntarily underwent whole-spine MRI. There were no abnormal findings in her mother, but surprisingly, an abnormal epidural band with lipomatosis dorsal to the ligament was observed in her father (Fig. 3A, B). However, the band did not compress the spinal cord.

## 4 Discussion

There are many reports demonstrating that epidural lipomatosis can cause myelopathy in the thoracic spine.^[2–4]^ Exogenous steroid use, obesity, and Cushing syndrome have been well recognized as frequent causes of spinal epidural lipomatosis. Our patient also had a large amount of epidural fat tissue deposition. So far, there are only 4 documented cases diagnosed as myelopathy secondary to fibrous epidural ligament (Table 1). Spinal cord compression was seen in the thoracic spine in all 4 patients. Zhou et al demonstrated that an abnormal epidural ligament is always accompanied by epidural lipomatosis.^[5]^ Three cases were initially misdiagnosed as intraspinal lipoma or angiolipoma and then required secondary operations. It has been reported that the abnormal ligament is often tightly adherent to the dural sac in adult cases. Fortunately, in our case, the fibrous epidural ligament could be easily separated from the dura mater. The degree of adhesion between the ligament and dura mater might differ in different pathologic stages. Therefore, the pathologic state of the abnormal ligament in our patient appeared to be a relatively early phase. A review of the 4 documented cases suggests that the earlier patients are diagnosed, the better the neurologic outcome. Thus, spine surgeons should be cognizant of the fact that this rare ligament typically coexists with epidural fat tissue deposition to enable an accurate diagnosis as early as possible.

**Table 1:**
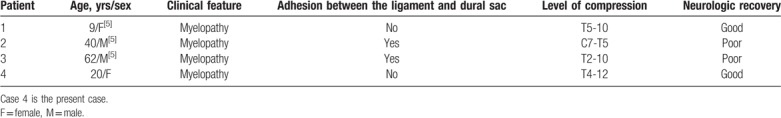
Summary of patient demographics, pathology, and treatment of the 4 cases reported in the literature to date.

Meningovertebral ligaments are often observed in a single vertebral segment. Hamid et al demonstrated that small ligaments become apparent after 39 weeks of gestation.^[6]^ Wadhwani et al suggested that fibrous connective tissue between the dural sac and surrounding structures, which are loose during fetal development, become reorganized into a series of fibrous bands after birth.^[7]^ Although the pathogenesis of fibrous epidural ligaments is still unclear, we speculate that embryogenic errors in the development of the dorsal meningovertebral ligaments could occur. The resulting morphologic changes in the spine with increasing age could account for ligament hypertrophy. The same abnormal fibrous ligament was observed in the patient's father, so the findings of this case report suggest that the embryogenic error could be inherited. In the future, pedigree analysis is necessary to identify whether this anatomical abnormality is an inherited genetic disorder.

## Acknowledgment

The authors acknowledge Nobuko Nakajima for data collection.

## Author contributions

**Conceptualization:** Takahiro Tanimoto.

**Data curation:** Takashi Hirai, Takahiro Tanimoto, Hiroyuki Inose.

**Formal analysis:** Takahiro Tanimoto.

**Investigation:** Shuta Ushio, Shinichi Sasaki, Hiroyuki Inose.

**Methodology:** Shuta Ushio, Shinichi Sasaki, Hiroyuki Inose, Masato Yuasa.

**Project administration:** Takashi Hirai, Toshitaka Yoshii, Shuta Ushio, Masato Yuasa.

**Supervision:** Toshitaka Yoshii, Shinichi Sasaki, Atsushi Okawa.

**Validation:** Atsushi Okawa.

**Visualization:** Masato Yuasa.

**Writing – original draft:** Takashi Hirai, Takahiro Tanimoto.

**Writing – review & editing:** Takashi Hirai, Atsushi Okawa.

## References

[1] Shi B Li X Li H The morphology and clinical significance of the dorsal meningovertebral ligaments in the lumbosacral epidural space. Spine (Phila Pa 1976) 2012;37:E1093–8. 22565391 10.1097/BRS.0b013e31825c05ea

[2] Akhaddar A Ennouali H Gazzaz M Idiopathic spinal epidural lipomatosis without obesity: a case with relapsing and remitting course. Spinal Cord 2008;46:243–4. 17607308 10.1038/sj.sc.3102099

[3] Kalia LV Lee L Kalia SK Thoracic myelopathy from coincident fluorosis and epidural lipomatosis. Can J Neurol Sci 2010;37:276–8. 20437944 10.1017/s0317167100010076

[4] Al-Yafeai R Maghrabi Y Malibary H Spinal cord compression secondary to idiopathic thoracic epidural lipomatosis in an adolescent: a case report and review of literature. Int J Surg Case Rep 2017;37:225–9. 28710985 10.1016/j.ijscr.2017.06.041 PMC5510523

[5] Zhou Y Chai X Zheng H Spinal cord compression syndrome caused by intraspinal epidural fibrous cord: three case reports. Medicine (Baltimore) 2017;96:e7592. 28816943 10.1097/MD.0000000000007592 PMC5571680

[6] Hamid M Fallet-Bianco C Delmas V The human lumbar anterior epidural space: morphological comparison in adult and fetal specimens. Surg Radiol Anat 2002;24:194–200. 12375072 10.1007/s00276-002-0041-6

[7] Wadhwani S Loughenbury P Soames R The anterior dural (Hofmann) ligaments. Spine (Phila Pa 1976) 2004;29:623–7. 15014271 10.1097/01.brs.0000115129.59484.24

